# Cofilin: A Promising Protein Implicated in Cancer Metastasis and Apoptosis

**DOI:** 10.3389/fcell.2021.599065

**Published:** 2021-02-04

**Authors:** Jing Xu, Yan Huang, Jimeng Zhao, Luyi Wu, Qin Qi, Yanan Liu, Guona Li, Jing Li, Huirong Liu, Huangan Wu

**Affiliations:** ^1^Yueyang Hospital of Integrative Chinese and Western Medicine, Shanghai University of Traditional Chinese Medicine, Shanghai, China; ^2^Key Laboratory of Acupuncture and Immunological Effects, Shanghai University of Traditional Chinese Medicine, Shanghai, China

**Keywords:** cofilin, actin-binding protein, apoptosis, migration, cancer metastasis

## Abstract

Cofilin is an actin-binding protein that regulates filament dynamics and depolymerization. The over-expression of cofilin is observed in various cancers, cofilin promotes cancer metastasis by regulating cytoskeletal reorganization, lamellipodium formation and epithelial-to-mesenchymal transition. Clinical treatment of cancer regarding cofilin has been explored in aspects of tumor cells apoptosis and cofilin related miRNAs. This review addresses the structure and phosphorylation of cofilin and describes recent findings regarding the function of cofilin in regulating cancer metastasis and apoptosis in tumor cells.

## Introduction

Actin-binding proteins are abundant cellular proteins that regulate cell function by mediating actin polymerization and remodeling (Dos Remedios et al., 2003; Virtanen and Vartiainen, 2017). Cofilin is an actin-binding protein and is function as a severing protein that severs actin filaments (Wang et al., 2007; Huang et al., 2014; Chang et al., 2015). Cofilin is known as a regulator of actin filament dynamics, it is a small protein of ~21 kDA that is ubiquitously expressed in all vertebrates and freely diffuses in eukaryotic cells (Shishkin et al., 2016). Cofilin promotes the conversion of actin filaments by enhancing the F-actin depolymerization and inhibiting the G-actin polymerization, which are essential in the actin filament dynamics of eukaryotes (Berger and Moeller, 2011). The phosphorylation and dephosphorylation of cofilin at the Ser^3^ site are crucial mechanisms for actin depolymerization and assembly. Once cofilin is activated by dephosphorylation, it servers actin by translocating into the nucleus with binding to actin (Ishikawa-Ankerhold et al., 2017). In recent decades, studies have reported that overexpression of cofilin is universal for cancer cells, regardless of the type of tumor, increased levels of cofilin is positively correlated with malignant phenotypes, as well as the cancer metastasis (Yang et al., 2020). Cancer metastasis involves tumor cell migration, which is a process requires cell motility to translocate tumor cells from the primary organ. Cofilin promotes the cell motility by regulating the cytoskeletal reorganization, promoting the lamellipodium formation, cell–cell adhesion dissolution, epithelial-to-mesenchymal transition (EMT) process and “migration-by-tethering” mechanism, thus participate in the cancer metastasis. As an important regulator of cancer metastasis, more and more studies explored the potential of cofilin being a therapeutic target in tumors. Activated cofilin translocates to the outer mitochondrial membrane and interacts with dynamin-related protein 1 (Drp1), induces mitochondrial fission and promotes cytochrome C release, finally leading to apoptosis in tumor cells (Hoffmann et al., 2019; Hu et al., 2020). This review discusses the functional role of cofilin in cancer metastasis and provides evidence for clinical perspective of cofilin in cancer treatment.

## Structure of Cofilin

**Figure 1:**
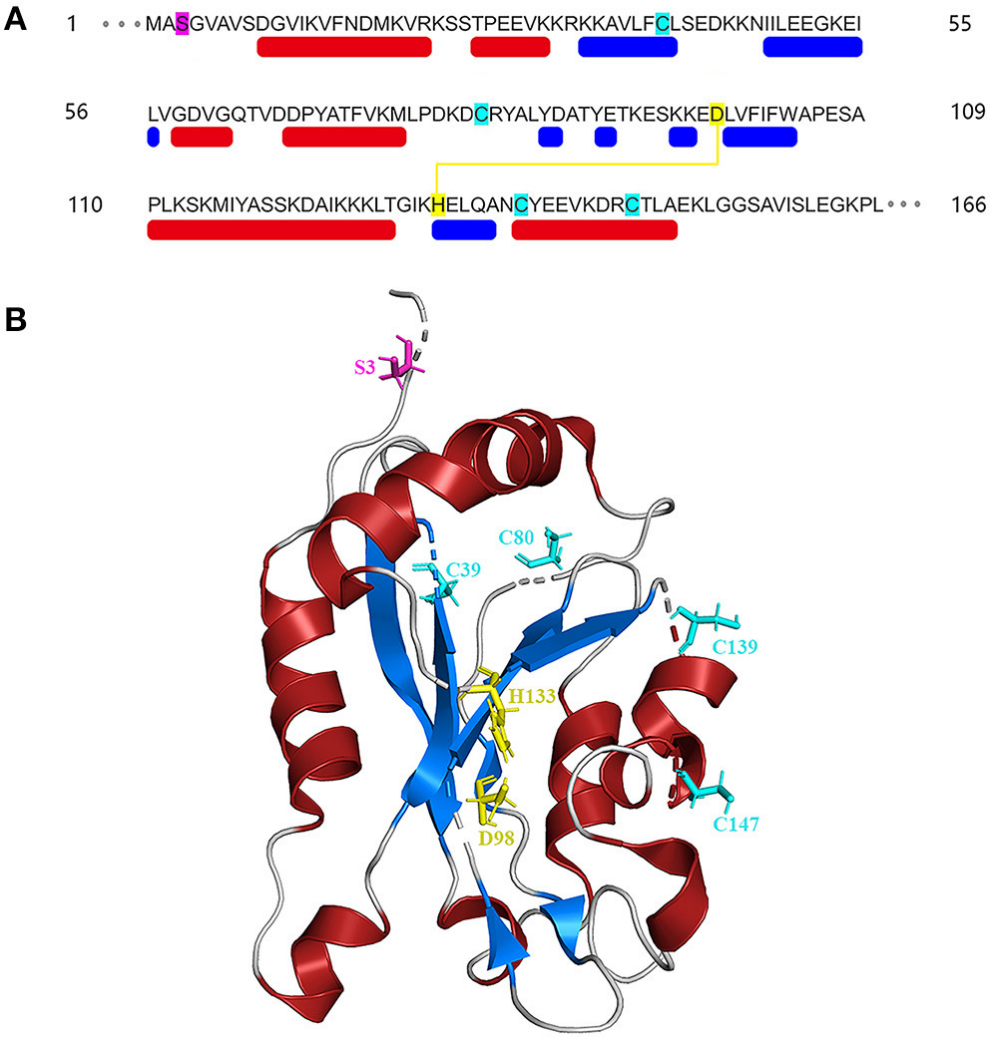
Structure of cofilin. **(A)** Sequence schematic of the secondary structural elements and binding sites of cofilin according to PDB (1Q8G). The red and blue boxes below the sequence correspond to α-helices and β-strands, respectively. Ser^3^ is labeled in magenta, Asp^98^ and His^133^ are labeled in yellow, and Cys^39^, Cys^80^, Cys^139^ and Cys^147^ are labeled in green. The yellow line indicates the salt bridge. **(B)** Ribbon diagrams of cofilin generated by PyMol. The α-helices and β-strands are shown in red and blue, respectively. The binding sites are shown in the same color as in **(A)**.

The amino acid sequences of cofilin consists two actin-binding sites, the F-site and the G/F-site. The F-site locates in the N-terminus, which is responsible for binding to F-actin and severing actin filaments. The G/F-site, locates in the C-terminus, binds to both G-actin and F-actin in the same ratio (Nishida et al., 1984; Lappalainen et al., 1998; Shukla et al., 2015). The sequence schematic and ribbon diagrams are shown in Figure 1. Activated cofilin dissociates subunits of actin filaments by translocating into the nucleus with binding to actin (Ishikawa-Ankerhold et al., 2017). Actin hydrolyzes ATP into ADP, cofilin binds to ADP-actin in the actin filaments, leads to the severing and dissociation of actin filaments (Carlier et al., 1997). During this process, free barbed ends are produced and turnover rate is increased, which promote the cyclic use of F-actin (Carlier et al., 1997; Bravo-Cordero et al., 2013; Hsiao et al., 2015). Several binding sites of cofilin exert essential effects on the regulation of cellular functions. The Asp^98^ and His^133^ sites of the cofilin protein build a salt bridge, and the construction of this bridge is especially correlated with the pH sensitivity and stabilization of the molecule structure (Pope et al., 2004). Amino acids 15–30 and amino acids 106–166 locates in N-terminus and C-terminus respectively, they are required for mitochondrial targeting, thus play a crucial role in pro-apoptotic function (Chua et al., 2003). Cys^39^, Cys^80^, Cys^139^and Cys^147^ are four important sites of the cofilin for oxidation-mediated regulation of mitochondrial translocation (Klamt et al., 2009). Phosphorylation/dephosphorylation of cofilin can be achieved through Ser^3^ site in combination with LIM domain kinase (LIMK) and slingshot phosphatases (SSH). Phosphorylation on Ser^3^ deactivate cofilin, while dephosphorylation works in the opposite way. In addition, the phosphorylation status of Ser^3^ also affects the ability of cofilin to translocate to the mitochondria (Chua et al., 2003; Kalendová et al., 2014). Dephosphorylated cofilin can translocate to the mitochondria and participate in the regulation of mitochondria-mediated apoptosis (Kalendová et al., 2014).

## Cofilin Phosphorylation/Dephosphorylation

**Figure 2:**
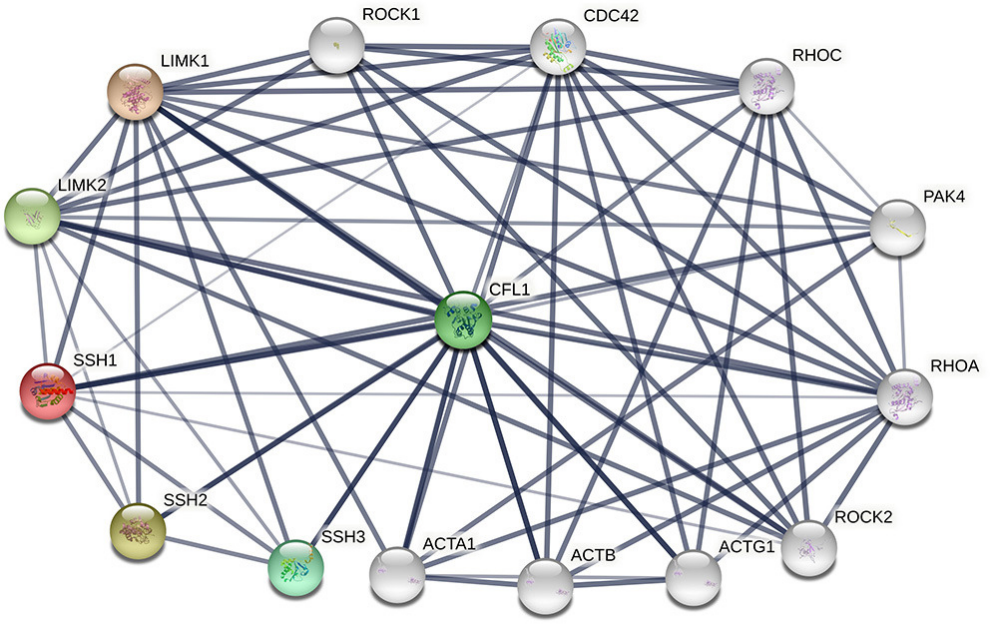
The protein-protein interactions (PPI) of cofilin, LIMKs and SSHs. Colored nodes represent query proteins and first shell of interactors, white nodes represent second shell of interactors, lines represent the interactions between two proteins.

The phosphorylation/dephosphorylation status determine the activity of cofilin, which is a key regulating mechanism of actin filament dynamics and cell motility, including actin cytoskeletal reorganization and cell-cell adhesion (Mizuno, 2013). Actin filament dynamics mainly refer to the coordinated assembly and disassembly of F-actin, which are responsible for the alteration of cytoskeletal structure (Etienne-Manneville and Hall, 2002). Phosphorylation on ser^3^ deactivate cofilin and release it from actin, thereby inhibit its ability to severing and depolymerizing F-actin, decreasing the cellular concentration of G-actin and consequently decreasing the turnover rate of actin filaments (Hotulainen et al., 2005; Kiuchi et al., 2007, 2011). The phosphorylation of cofilin is regulated by activated LIM kinases (LIMK1 and LIMK2), LIMK is a kinase that includes two main isoforms. LIMK1 is expressed mostly in the parathyroid gland, cerebral cortex, bronchus and stomach, while LIMK2 is highly expressed in the thyroid gland, smooth muscle, pancreas, testis, and ovaries (Po'uha et al., 2010; Mardilovich et al., 2015). LIMKs can be activated by phosphorylation, ROCK, PAK1, PAK2, PAK4 and MRCKα are regulators that reduce LIMK phosphorylation by binding to Thr^508^ (LIMK1) and Thr^505^ (LIMK2) threonine residue, whereas upstream effectors are Rho GTPases, including RhoA, Rac1, and Cdc42 (Mizuno, 2013). Therefore, Rho GTPase pathway is essential for cofilin phosphorylation. The dephosphorylation of cofilin is regulated by SSH phosphatase, SSH1, SSH2, and SSH3 are three isoforms of SSH, all SSHs efficiently dephosphorylate cofilin and counteract aberrant F-actin assembly (Niwa et al., 2002; Ohta et al., 2003), although the effect of SSH3 dephosphorylating cofilin is weaker than SSH1 and SSH2. SSH1, SSH2, and SSH3 have different subcellular distributions, and their expression patterns in different tissues are different, indicating that these three isoforms may have unique mechanisms by which they participate in cellular and biological functions (Niwa et al., 2002; Ohta et al., 2003). SSH increases the level of dephosphorylated cofilin on Ser^3^, activates the cofilin ability of binding to actin and severing F-actin, resulting in the depolymerization of F-actin and increasing of actin turnover rate. This is an important mechanism for the formation and extension of F-actin-rich lamellipodium at the leading edge of the cell, which is responsible for polarized cell mobility. In addition, SSH1 can inhibit the LIMKs phosphorylation activity toward cofilin by dephosphorylating them (Soosairajah et al., 2005), indicated that SSH1 activates cofilin not only by dephosphorylating cofilin, but also by suppressing the LIMK/cofilin activity. Overall, LIMK and SSH are two important regulators of cofilin, can bind to cofilin at Ser^3^ and regulate the activity of cofilin and the invasion ability of cells (Ivanovska et al., 2013). Protein-protein interaction (PPI) enrichment shows that cofilin, LIMK and SSH are strongly correlated with each other, the interactome map constructed by the 10 most significant correlated proteins around cofilin, LIMK and SSH is shown in Figure 2. ROHA, RHOC, ROCK1, ROCK2, and PAK4 are upstream effectors of LIMKs in Rho GTPase signaling pathway, which can indirectly promote the phosphorylation of cofilin and inhibit the activated cofilin activity of depolymerizing F-actin, thus can stabilize the actin cytoskeleton. On the contrast, decreasing of phosphorylated cofilin related to increasing of actin turnover. Mutations of SSH loss the function of dephosphorylating cofilin, resulting in a large increase of P-cofilin level and F-actin in cells, which is a similar phenomenon induced by LIMK. In this case, LIMK and SSH are considered to act in two opposite directions and are essential for the balance of the phosphorylation and dephosphorylation of cofilin, dephosphorylated cofilin is considered as activated cofilin and is necessary for severing F-actin, but phosphorylation is equally important as a prerequisite of binding and severing F-actin for this process can release the cofilin from filaments. Therefore, LIMK and SSH work together mediating the phosphorylation/dephosphorylation status of cofilin, this is essential for cofilin to function properly as it maintains the dynamic balance between actin polymerization and actin turnover rate, thus affects the pool of G-actin and F-actin (Jovceva et al., 2007; Scott et al., 2010). However, the activity of SSH and LIMK is not always opposite. SSH1 can also stabilize F-actin from cofilin-induced depolymerization and severing (Kurita et al., 2007), suggested that activation of SSH1 may alter its function dramatically, activated SSH1 depolymerizes F-actin by phosphatase cofilin, while inactivated SSH1 stabilizes F-actin-bundling. LIMK also participates in assembly of new actin filaments by severing F-actin in collaboration with actomyosin contraction via RhoA/ROCK pathway (Wang and Townes-Anderson, 2015).

## Cofilin in Cancers

### Cofilin as a Potential Biomarker of Cancers

The mRNA levels and expression of cofilin were significantly increased in tumor tissues than in benign prostatic hyperplasia tissues or normal tissues, this was a common phenomenon that observed in various types of cancer, such as hepatoblastoma (Liu et al., 2018), breast cancer (Maimaiti et al., 2017), non-small cell lung cancer (Wei et al., 2012), prostate cancer (Collazo et al., 2014; Lu et al., 2015), colorectal cancer (Sousa-Squiavinato et al., 2019), vulvar squamous cell carcinoma (Wu et al., 2016), ovarian cancer (Chen et al., 2014), and bladder cancer (Wang et al., 2017). The overexpression of cofilin may be closely related to the proliferation, invasion, and migration of cancers (Wang et al., 2007; Bernstein and Bamburg, 2010; Bravo-Cordero et al., 2013; Chang et al., 2015). High expression of cofilin was found to be positively correlated with dedifferentiation, lymphatic metastasis (Lu et al., 2015; Wu et al., 2016), haematogenous dissemination of tumors (Satoh et al., 2017) and shorter overall survival (Maimaiti et al., 2017). However, tumor size, pathological stage and patient age were not found to be associated with the expression of cofilin (Lu et al., 2015; Maimaiti et al., 2017). In *in vitro* experiments, when cofilin was knocked down, the growth and chemotaxis of tumor cells were significantly decreased; in addition, the cells were arrested in the G1 phase of the cell cycle, lamellipodium formation was disrupted, and invasion and metastasis were reduced (Wu et al., 2016). In addition to the overexpression of cofilin in many kinds of cancers, one study found that dephosphorylated cofilin expression in breast cancer tissues predicted lower overall survival, suggested that the dephosphorylated cofilin expression, other than the overall cofilin expression, can affect breast cancer prognosis (Maimaiti et al., 2016). Another study found that the cofilin immune complexes levels were significantly higher in pancreatic ductal adenocarcinoma patients than in healthy controls (Satoh et al., 2017). These results suggested that cofilin, including cofilin immune complexes, is a potential diagnostic tumor biomarker, it can be a therapeutic target and prognosis indicator of cancers.

### Cofilin Regulates the Cancer Metastasis

Multiple studies have verified that inhibition or enhancement of cofilin expression can make significant differences in tumor cell dynamics, thus influence the cancer metastasis. Cancer metastasis is a progress involving tumor cell migration into lymph nodes or blood vessels (Nieto et al., 2016). Tumor cell migration can be promoted by the formation of lamellipodium, constantly and repeatedly pulling the posterior cell forward under the action of cell contractility (Aung et al., 2014; Dalaka et al., 2020). Dephosphorylation of cofilin by SSH can induce the lamellipodium formation and extension, thus affecting the morphology, polarity and movement direction of cells (Chan et al., 2009). Cofilin is an indispensable controller of lamellipodium formation (Shishkin et al., 2016). Dephosphorylated cofilin promotes actin cytoskeleton reorganization by depolymerizing F-actin, stimulates actin turnover and, which augments the lamellipodium formation and extension, promote the cancer metastasis (Ghosh et al., 2004; Chan et al., 2009; Bravo-Cordero et al., 2013). Cofilin mediated lamellipodium formation and lamellipodium related cellular mobilization can be inhibited by the activation of JNK/Bnip3/SERCA/CaMKII pathways, therefore suppress the hepatocellular carcinoma metastasis. Phosphorylated JNK contributed to Bnip3 expression. Higher Bnip3 contributed to ATP undersupply (Fuhrmann and Brune, 2017). The energy disorder blunted the ability of SERC, leading to the activation of CaMKII (Hu et al., 2017). CaMKII can inhibit the F-actin assembly and lamellipodium formation by phosphorylating cofilin, eventually limiting the cancer migration (Zhang et al., 2016). SSH can be recruited to the lamellipodium and activated by F-actin, leads to the dephosphorylation of cofilin in the lamellipodium (Kurita et al., 2008). Inactivation of SSH1 inhibits the dephosphorylation of cofilin, limit actin cytoskeleton reorganization and lamellipodium formation, suppress the metastasis of cancer (Peterburs et al., 2009; Zhuang et al., 2018). Upregulation of SSH1 increases tumor cell migration in pancreatic cancer (Wang et al., 2015). Phosphorylation of cofilin regulated by LIMK can abrogate actin depolymerization activities and enhances stabilization of actin filament, inhibits the lamellipodium formation and tumor cell mobilization (Wioland et al., 2017). The overexpression of LIMK1 phosphorylated cofilin and supressed the cancer metastasis by suppressing of lamellipodium formation, while mutated LIMK1 increases the motility of tumor cells (Meyer et al., 2005; Li Z. et al., 2014), similar results were observed in studies of LIMK2 (Collazo et al., 2014; Xu et al., 2019). Nonetheless, conflicting results have been observed regarding the role of LIMK. LIMK1 overexpression promoted the cancer progression (Tania et al., 2013), while knockdown of LIMK1 inhibits the lamellipodium formation and reduced tumor cell migration (Nakashima et al., 2005; Chen et al., 2014). These contrary results indicated that although LIMK and SSH phosphorylate and dephosphorylate cofilin respectively, they don't necessarily work in an opposite way. Mathematical simulations suggested that LIMK-dependent cofilin phosphorylation also participates in assembly of new actin filaments, phosphorylated cofilin releases from filaments, which allows cofilin ready to bind and sever other filaments (Bravo-Cordero et al., 2013). Activation of RhoA-ROCK-MLC/MLCP pathway promote severing of actin filaments in collaboration with actomyosin contraction through cofilin activity (Wang and Townes-Anderson, 2015). LIMK promote polymerization of actin, which contributes to the formation of new actin branches and extension of actin meshwork, this process drives membrane forward at the leading edge. Overall, the dynamic balance between phosphorylation and dephosphorylation of cofilin is the key to F-actin homeostasis, LIMK and SSH are two important effectors of cofilin activity, dysfunction of LIMK or SSH would break this balance and lead to pathological changes, such as lamellipodium formation and cancer metastasis.

Moreover, cofilin can promote the cancer metastasis by regulating epithelial-to-mesenchymal transition (EMT). EMT can dissolve cell–cell adhesion and alter the cell morphology to fibroblast-like forms as a consequence of actin reorganization, which collectively translate into metastasis properties (Chaffer et al., 2016; Derynck and Weinberg, 2019; Yang et al., 2020). Cofilin is a terminal effector of Rho GTPase signaling, which is a major pathway of the actin cytoskeleton dynamics. Moreover, Rho GTPases are responsible for the formation of cell-cell adhesion and stabilization of adhesion (Anastasiadis and Reynolds, 2001). One study found a prominent accumulation of F-actin in EMT tumor cells, knockdown of cofilin abolished the morphologic pattern in EMT tumor cells. This result indicated that the EMT process in tumor cells may be regulated by phosphorylation of cofilin via Rho GTPase signaling (Haga and Ridley, 2016; Sousa-Squiavinato et al., 2019). Rho/ROCK/LIMK/cofilin is one of the Rho GTPase pathways, the inhibition of Rho/ROCK/LIMK/cofilin pathway resulted in the destroy of F-actin stabilization and redistribution of cytoplasmic actin via inhibition of cofilin phosphorylation, which promoted EMT process as well as gastric cancer metastasis. RICS and PRP4 are two GTPase-activating proteins that directly interacts with Rho, they function as upstream effectors and inhibit phosphorylation of cofilin by inactivate LIMK (Islam et al., 2018; Xu et al., 2020). However, another cofilin related pathway showed opposite relationship between phosphorylation of cofilin and EMT process. The inhibition of the Src/Akt/mTOR/cofilin pathway impaired the organization of actin cytoskeleton and suppress the EMT in melanoma cells via phosphorylation of cofilin. These results suggested that apart from phosphorylation/phosphorylation of cofilin, the breaking balance of phosphorylated and non-phosphorylated cofilin may be the key to changes in the dynamics of the actin cytoskeleton and EMT process of tumor cells (Wang et al., 2006). In addition, PRP4 can mediate the EMT by increasing the expression of PP1A other than cofilin. PP1A induces dephosphorylation of MIIP, resulting in the down-regulation of E-cadherin protein levels, which further promote the process of EMT (Islam et al., 2018). This might be another reason of the opposite results.

In addition to promoting EMT and lamellipodium formation, there are other potential mechanisms of cofilin participate in cancer metastasis. The mechanically rigid tissue surrounding a tumor is denser compared to normal tissue, and increased rigidity of substrates can enhance tumor cell migration (Tlsty and Coussens, 2006). Mechanical stimuli (tension) can trigger a mechanical response pathway in normal fibroblasts, resulting in increasing amount of fibronectin in the substrates (Kostic and Sheetz, 2006; Friedland et al., 2009). Mechanical stimuli (tension) can induce the decreasing of actin twist angle and change the filaments structural, increase the ratio of filament stiffness (Matsushita et al., 2011). Tension triggers a mechanical response pathway in normal fibroblasts, resulting in increasing amount of fibronectin in the substrates (Kostic and Sheetz, 2006; Friedland et al., 2009). During this process, cofilin plays a crucial role (Hayakawa et al., 2011). Mechanical stimuli (tension) can be directly sensed by actin filaments and induce changes in the filament dynamics, which decreases the binding rate of cofilin to F-actin, leading to an inhibition of the severing activity of cofilin. Cofilin preferentially binds to flexible twisted F-actin, when tension in the filament is increased by stretch, the magnitude of torsional fluctuations of the filament will be reduced, resulting in an inhibition of cofilin interaction with F-actin (Hayakawa et al., 2011; Matsushita et al., 2011). In *in vitro* experiment, the invasion of stimulated tumor cells are higher than non-stimulated cells, but the invasion between stimulated or non-stimulated tumor cells was not significant different when cofilin was silenced, indicated that cofilin is needed in the tension induced tumor cell migration (Menon and Beningo, 2011). Migration-by-tethering is a mechanism proposed recently. This mechanism was observed and explored in breast cancer, dendritic spine-like structure (DSLS) narrows the distance between tumor cells and osteogenic cells, thus increases the mobility of the otherwise inert tumor cells. DSLS is the key to migration-by-tethering, it is abundant with cofilin, thereby it has high flexibility and cell adhesion, this ability allows DSLS to combine with osteogenic cells through cell-cell adhesion, such as adherheterotypic adherens junctions and gap junctions. This process can drive cancer cells that do not possess intrinsic migratory properties to acquire the ability of migration (Muscarella et al., 2020).

## Clinical Perspective of Cofilin in Cancer Treatment

### Cofilin Is Involved in Regulating Apoptosis in Tumor Cells

Apoptosis is an active, controlled and complicated process, it is the degradation of a highly conserved protein or organelle in eukaryotes (Shi et al., 2020). Allyl isothiocyanate (AITC) (Tang et al., 2014), urisolic acid (UA) (Li R. et al., 2014), etoposide (Chua et al., 2003), arnidiol (Hu et al., 2020), and 4-methylthiobutyl isothiocyanate (Grzanka et al., 2011) can induce apoptosis in several tumor cell lines, such as SH-SY5Y, HL60, COS-7, and HeLa cells (Chua et al., 2003), through the cofilin pathway by regulating mitochondrial translocation and fission (Hoffmann et al., 2019; Hu et al., 2020). The fusion and division of mitochondria are two continuous dynamic antagonistic processes, which maintain the morphology of mitochondria and apoptotic fission plays essential role in cellular physiology (Sheridan and Martin, 2010). Cofilin involves in the process of mitochondrial fission (Hatch et al., 2014; Li et al., 2015). ROCK1/PTEN/PI3K signaling pathway is the first step in mitochondrial division (Wang et al., 2012; Li et al., 2013). Activated ROCK1 is the upstream protein that directly regulates PTEN (Di Cristofano and Pandolfi, 2000; Yan and Backer, 2007; Li R. et al., 2014). The activation of ROCK1 leads to the activation of PTEN, resulting in the inhibition of PI3K activity (Vasudevan et al., 2011). PI3K is the upstream molecule that directly regulates PP1/PP2A (Bamburg and Bernstein, 2016). Inhibition of PI3K activity will inhibit the dephosphorylation of Akt in PI3K pathway, increase PP1/PP2A activity, and lead to the increase of Cofilin phosphorylation (Song et al., 2015). PP1/PP2A is a direct upstream regulator of Cofilin dephosphorylation activation (Ambach et al., 2000; Eichhorn et al., 2009). Increased expression of PP1/PP2A phosphatase can promote cofilin dephosphorylation activation (Delorme-Walker et al., 2015). Then the dephosphorylated cofilin translocates to the outer membrane of the mitochondria to bind directly to F-actin, and depolymerize the F-actin into G-actin, causing the mitosis of the mitochondria. The transient mitochondrial assembly of F-actin is vital for mitochondrial fission, it ensures the smooth progress of the dynamic cycle of F-actin/G-actin in the process of mitochondrial division, and thus participates in the regulation of mitochondrial division (Chen et al., 2000; Li et al., 2015). Subsequently, mitochondrial damage and cytochrome C release lead to the degradation and activation of Capase-9 and Capase-3, and finally lead to apoptosis (Morley et al., 2003). One study showed that inhibition of the Src/Akt/mTOR signaling pathway resulted in decreased levels of dephosphorylation of cofilin (Li et al., 2019), this indicates that Src/Akt/mTOR signaling pathway may be another upstream signaling pathway activated by cofilin.

**Figure 3:**
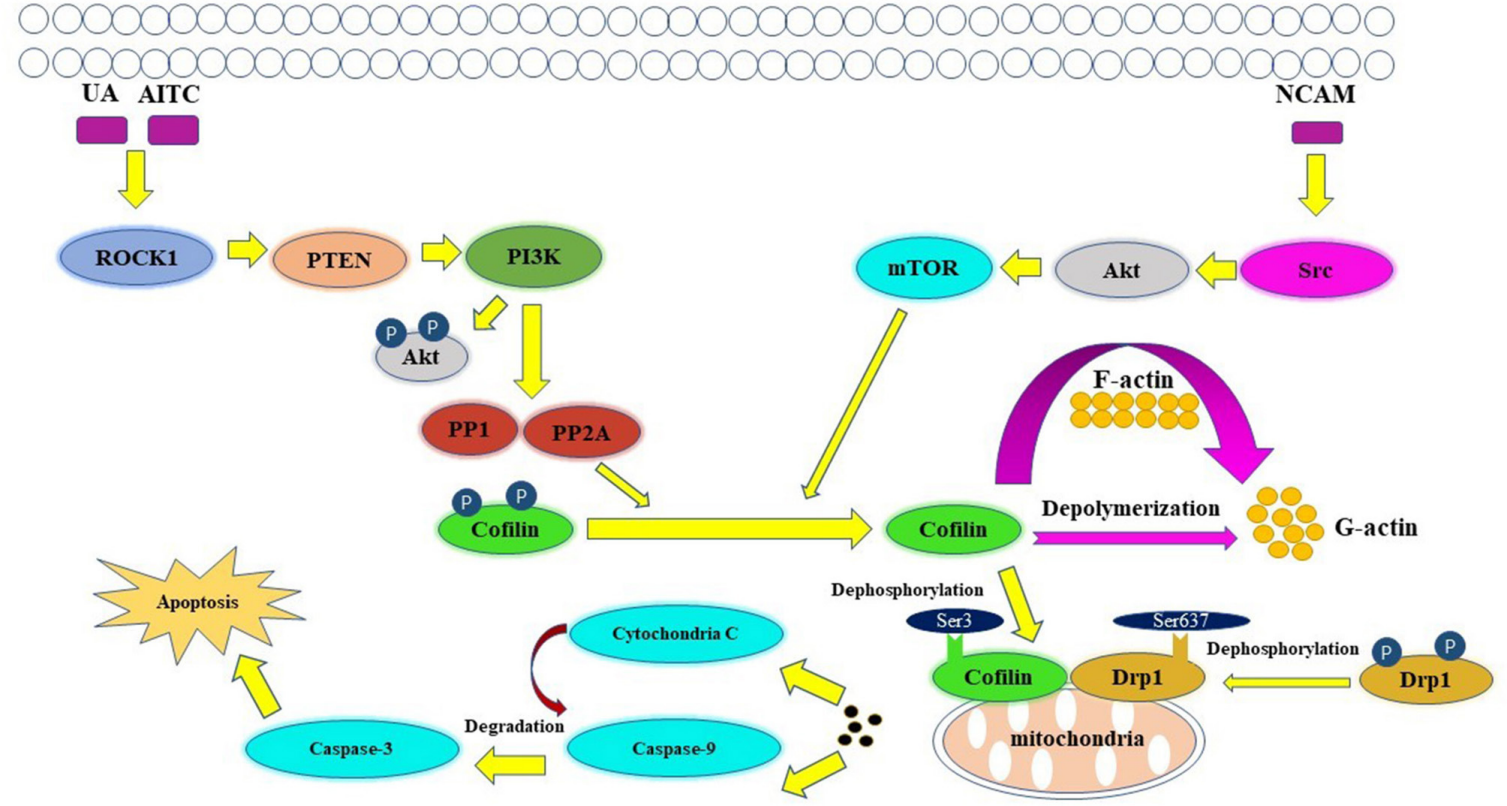
Key figure showing a schematic of cofilin-mediated apoptosis (Li et al., 2013). UA, AITC, etoposide and arnidiol induce apoptosis through the cofilin pathway. Activated cofilin translocates to the outer mitochondrial membrane and interacts with Drp1, induces mitochondrial fission and promotes cytochrome C release, finally leading to apoptosis.

The mitochondrial regulation dominated by cofilin dephosphorylation activation is closely related to Drp1 and PINK1/Park2 pathways (Serasinghe and Chipuk, 2017). The direct interaction between cofilin and Drp1 in the outer membrane of mitochondria contributes to mitochondrial division (Estaquier and Arnoult, 2007; Hu et al., 2020). Knocking down the expression of cofilin or Drp1 will affect their interaction, resulting in the blocking of mitochondria division and the release of cytochrome C and apoptosis (Li et al., 2015; Rehklau et al., 2017). The dephosphorylation status of cofilin Ser^3^ site and the dephosphorylation of Drp1 Ser^637^ site are key sites of cofilin-Drp1-mediated mitochondrial damage (Chua et al., 2003; Archer, 2013; Bamburg and Bernstein, 2016). The dephosphorylated activated plasmid cofilin (S3A) could induce the increase of cofilin mitochondrial translocation, leads to the increase of mitochondrial division and induce cell apoptosis, while its phosphorylated inhibitory plasmid cofilin (S3E) could induce the decrease of mitochondrial translocation and block mitochondrial division, resulting in the decrease of apoptosis (Hu et al., 2020). The dephosphorylated activated plasmid Drp1 (S637A) can induce the increase of Drp1 mitochondrial translocation, which leads to the increase of mitochondrial division and apoptosis; while the phosphorylated inhibitory plasmid Drp1 (S637D) reduces the mitochondrial translocation of Drp1 and inhibits mitochondrial division resulting in the decrease of apoptosis (Hu et al., 2020). Drp1, which is a hydrolytic GTP enzyme, is a key molecule in regulating mitochondrial division in mammalian cells (Rehklau et al., 2017). In the early stage of apoptosis, Drp1 protein can be dephosphorylated and activated and translocated to the mitochondrial outer membrane together with dephosphorylated cofilin (De Vos et al., 2005; Ji et al., 2015). Cofilin binds directly to the potential mitotic site of mitochondria and wraps the mitochondria to form a circular complex that can regulate mitochondrial division (Satoh et al., 2017). The distance or angle between molecules is changed by GTP hydrolysis of Drp1, then contracting the Drp1 ring gradually and constricting the mitochondria, and then cause mitochondrial damage by regulating the division of the mitochondria (Frank et al., 2001; Wang et al., 2009). At the same time, mitochondrial division is accompanied by PINK1/Park2 pathway mitochondrial autophagy (Greene et al., 2012; Ashrafi and Schwarz, 2013; de Vries and Przedborski, 2013). PINK1/Park2 pathway is the key pathway to regulate mitochondrial autophagy (Jin et al., 2010; Springer and Kahle, 2011). Cofilin can regulate mitochondrial autophagy mediated by PINK1/Park2 pathway by affecting mitochondrial membrane potential (Narendra et al., 2010; Fedorowicz et al., 2014). The expression of cofilin can induce mitochondrial division, down-regulate the mitochondrial membrane potential, further aggravate the down-regulation of the expression of MPP β, PARL and AFG3L2, lead to the activation of PINK1, increase the mitochondrial translocation of Park2 and the occurrence of mitochondrial autophagy (Li et al., 2018). A schematic of cofilin-mediated apoptosis is shown in Figure 3.

### MicroRNAs as Inhibitors of Cofilin Activity

MicroRNAs (miRNAs), such as miR-342, miR-429, miR-182-5p, act as inhibitors of cofilin activity and upstream effectors of proliferation and migration in cancer cells (Lowe and Lin, 2000; Lin et al., 2010; Tian et al., 2015; Liu et al., 2020). Other miRNAs, such as miR-138 and miR-384, modulate the activity and expression of cofilin through the LIMK/cofilin pathway (Chen et al., 2014; Yu et al., 2019). MiRNAs are non-coding RNAs that can suppress mRNA translation and inhibit protein activity by binding to the 3′UTR of their target mRNAs (Bartel, 2004; Ozols, 2005). MiRNAs are known to be tumor suppressors and are considered as therapeutic targets, they play crucial roles in various cellular processes that are closely related to tumor progression, overexpression of miRNAs significantly inhibit the proliferation (Hatfield et al., 2005; Garzon et al., 2010; Hayes et al., 2014; Su et al., 2015), dedifferentiation and migration of cancer cells (Lowe and Lin, 2000; Tian et al., 2015; Liu et al., 2020). Downregulation of miRNAs and overexpression of cofilin have been observed in different types of cancers, which may be closely related to the overexpression of oncogenes (Zhou et al., 2013; Chen et al., 2014; Tian et al., 2015; Yu et al., 2019; Liu et al., 2020). MiR-342 acts as an upstream effector of cofilin in human breast cancer cells, miR-429 targets cofilin in colon cancer cells; miR-182-5p binds to the 3′UTR of cofilin mRNA at position 135–142 in human bladder cancer cells (Lowe and Lin, 2000; Tian et al., 2015; Liu et al., 2020). Cofilin expression can be downregulated or upregulated due to the transfection-mediated overexpression or inhibition of these miRNAs, respectively (Lowe and Lin, 2000; Tian et al., 2015; Liu et al., 2020). Cancer cells transfected with anti-miRNAs can be rendered more invasive by promoting cofilin activity. These results indicated that miRNAs mediate the cancer metastasis by regulating the activity of cofilin (Tian et al., 2015). Some other miRNAs can indirectly affect the activity of cofilin by regulating the LIMK1/cofilin signaling pathway, and upregulation of certain miRNAs inhibits the levels of LIMK and vice versa. MiR-138 supressed the cancer metastasis by targeting LIMK1. Further experiment founds that cofilin participated in the inhibitory effect of miR-138 regulating tumor cells. Although LIMK1 was upregulated within knockdown miR-138 in cofilin knockout stable cell lines, the migration and invasion ability of tumor cells were not sufficiently promoted (Chen et al., 2014). MiR-384 affects cofilin activity by targeting LIMK1, thus modulating the progression of esophageal squamous cell carcinoma (Yu et al., 2019). These findings suggest that miRNAs act as promising inhibitors of cancer metastasis by inhibiting LIMK1/cofilin signaling activity.

## Conclusion

Cofilin is an actin-binding protein that is expressed in all kinds of mammals. Great progress has been made in understanding the structural function and biological effects of cofilin, and its effects on tumor development have been well-studied. Cofilin was found to be the major protein in different human cancer cells that can modulate cellular morphology, mitosis and mitochondrial fission. Cofilin plays an essential role in the cancer metastasis and apoptosis of tumor cells and is considered a promising biomarker of different cancers. The balance of kinases (LIMK1) and phosphatases (SSH1) can change the activation of cofilin, and LIMK1 and SSH1 have been extensively studied as regulators in cofilin-mediated pathways in cell motility and cancer metastasis. However, there are contradictory results and data regarding the expression of cofilin in tumor cells, effects of dephosphorylation of cofilin and the expression level of LIMK1 on cell migration and invasion. Further studies are needed to explore the potential mechanisms behind these contradictory results. The effect of cofilin on apoptosis is a new focus of studies on tumor development, and a growing body of research has found that cofilin is involved in apoptosis under the regulation of AITC, UA, etoposide and arnidiol in various leukemia cells and breast cancer cells. Active (dephosphorylated) cofilin induces apoptosis by translocating to the outer membrane of mitochondria and promoting the release of cytochrome C. Therefore, cofilin can be developed as a new anti-tumor target. The regulation of apoptosis by cofilin in cancer cells can be a very promising research direction. With further study of the pathway linking cofilin and apoptosis, cofilin may be not only a biomarker and prognostic indicator of cancers but also a therapeutic target for various cancers.

## Author Contributions

JX, YH, and JZ: wrote-original draft preparation. LW, QQ, YL, GL, and JL: wrote-review and editing. HL and HW supervised and approved the final submitted version. All authors contributed to the article and approved the submitted version.

## Conflict of Interest

The authors declare that the research was conducted in the absence of any commercial or financial relationships that could be construed as a potential conflict of interest.
